# Oral phytate supplementation on the progression of mild cognitive impairment, brain iron deposition and diabetic retinopathy in patients with type 2 diabetes: a concept paper for a randomized double blind placebo controlled trial (the PHYND trial)

**DOI:** 10.3389/fendo.2024.1332237

**Published:** 2024-05-30

**Authors:** Antelm Pujol, Pilar Sanchis, María I. Tamayo, Joana Nicolau, Félix Grases, Ana Espino, Ana Estremera, Elena Rigo, Guillermo J. Amengual, Manuel Rodríguez, José L. Ribes, Isabel Gomila, Olga Simó-Servat, Lluís Masmiquel

**Affiliations:** ^1^ Vascular and Metabolic Diseases Research Group, Endocrinology Department, Son Llàtzer University Hospital, Health Research Institute of the Balearic Islands (IdISBa), Palma de Mallorca, Spain; ^2^ Laboratory of Renal Lithiasis Research, University of Balearic Islands, Research Institute of Health Science (IUNICS) Health Research Institute of Balearic Islands, (IdISBa), Palma de Mallorca, Spain; ^3^ Neurology Department, Son Llàtzer University Hospital, Palma de Mallorca, Spain; ^4^ Neuroradiology Unit, Son Llàtzer University Hospital, Palma de Mallorca, Spain; ^5^ Neuroopthalmology Unit, Son Llàtzer University Hospital, Palma de Mallorca, Spain; ^6^ Biochemistry Department, Son Llàtzer University Hospital, Palma de Mallorca, Spain; ^7^ Diabetes and Metabolism Research Unit, Vall d’Hebron Research Institute, Universitat Autònoma de Barcelona, Barcelona, Spain

**Keywords:** type 2 diabetes mellitus, cognitive impairment, phytate, Mediterranean diet, mild cognitive impairment

## Abstract

Type 2 diabetes mellitus has a worldwide prevalence of 10.5% in the adult population (20–79 years), and by 2045, the prevalence is expected to keep rising to one in eight adults living with diabetes. Mild cognitive impairment has a global prevalence of 19.7% in adults aged 50 years. Both conditions have shown a concerning increase in prevalence rates over the past 10 years, highlighting a growing public health challenge. Future forecasts indicate that the prevalence of dementia (no estimations done for individuals with mild cognitive impairment) is expected to nearly triple by 2050. Type 2 diabetes mellitus is a risk factor for the development of cognitive impairment, and such impairment increase the likelihood of poor glycemic/metabolic control. High phytate intake has been shown to be a protective factor against the development of cognitive impairment in observational studies. Diary phytate intake might reduce the micro- and macrovascular complications of patients with type 2 diabetes mellitus through different mechanisms. We describe the protocol of the first trial (the PHYND trial) that evaluate the effect of daily phytate supplementation over 56 weeks with a two-arm double-blind placebo-controlled study on the progression of mild cognitive impairment, cerebral iron deposition, and retinal involvement in patients with type 2 diabetes mellitus. Our hypothesis proposes that phytate, by inhibiting advanced glycation end product formation and chelating transition metals, will improve cognitive function and attenuate the progression from Mild Cognitive Impairment to dementia in individuals with type 2 diabetes mellitus and mild cognitive impairment. Additionally, we predict that phytate will reduce iron accumulation in the central nervous system, mitigate neurodegenerative changes in both the central nervous system and retina, and induce alterations in biochemical markers associated with neurodegeneration.

## 1 Introduction

The treatment and prevention of increasing numbers of patients with type 2 diabetes mellitus (T2DM) and mild cognitive impairment (MCI) due to demographic and lifestyle changes are important challenges faced by healthcare systems. MCI is a milder form of cognitive dysfunction that precedes dementia and affects almost one in two people over the age of 65 with T2DM (1). People with MCI are at high risk of dementia (DE); the majority of studies indicate a progression rate to DE ranging from 20% to 40%, with an annual rate ranging between 5% and 17% (2). In addition, people with T2DM have a very high risk of MCI in comparation to the non-diabetic population. In these sense, it has been recognized that T2DM acts as an important accelerator of DE in patients with MCI (3, 4). Cognitive impairment makes self-care difficult and increases the risk of hypoglycemia, which increases comorbidity, hospital admissions, and the costs of T2DM to healthcare systems (5, 6).

T2DM has been correlated with an accelerated decline in cognitive function among the elderly and an increased likelihood of developing mild cognitive impairment and a higher risk of dementia, including both Alzheimer’s disease (AD) and vascular dementia (7). In fact, T2DM doubles the risk of developing AD. This increase is maintained even after adjusting for vascular risk factors (8, 9). The mechanism connecting diabetes to AD is multifactorial and not well-defined. However, its pathogenesis is known to involve a decrease in brain-derived neurotrophic factor (BDNF) and an increase in oxidative stress due to the accumulation of advanced non-enzymatic glycation products (AGEs), systemic inflammation via tumor necrosis alpha (TNF-a), interleukin 1beta (IL-1b) and interleukin 6 (IL-6), increased lipid peroxidation, and decreased superoxide dismutase (SOD) activity (7, 10). In AD, it has been seen that AGEs, through their receptor (RAGE), increase the expression of tau and Aß (11).

Concomitantly, people with T2DM have a higher prevalence of sarcopenia and dynapenia in comparison with an age-matched population without T2DM. This prevalence could be a consequence of chronic inflammation and oxidative stress associated with this condition (12). In addition, sarcopenia and dynapenia have been associated with cognitive impairment (13). The preservation of the muscle tissue could be an effective strategy for improving not only metabolic state but also cognitive performance.

Investigating the relationship between iron homeostasis and mild cognitive impairment (MCI) is pivotal, considering the emerging evidence suggesting a potential link between disrupted iron metabolism and cognitive decline. Iron plays a crucial role in various cellular processes, including oxygen transport, energy metabolism, and neurotransmitter synthesis. However, dysregulation of iron homeostasis can lead to the accumulation of excess iron in the brain, contributing to oxidative stress, neuroinflammation, and neuronal damage (14). Ferroptosis is a unique form of iron-mediated programmed cell, which is distinct from apoptosis, necrosis, autophagy, and other forms of cell death (14). Iron deposits in neurons are closely tied to neurodegeneration and cognitive decline, although the specific mechanisms driving these connections remain uncertain (14). Various factors can alter iron homeostasis, causing the gradual accumulation of iron in various tissues, including the central nervous system (CNS) (15–17). Aging itself and diseases such as AD, Parkinson’s disease, and Huntington’s disease, among others, are related to an increase in iron levels in the region of the brain affected. The level of iron accumulation also correlates with the severity of the disease (18, 19). In addition, iron overload is associated with age-related macular degeneration and a subset of psychiatric diseases (20, 21).

Ferroptosis plays a role in neuronal loss during acute or chronic brain injury, and blocking ferroptosis could decrease cell death and improve neurological function in animal models (22). Conservative iron chelation has been proposed as a new therapeutic concept for the treatment of neurodegenerative diseases associated with increased iron storage (21). Unlike the chelators used for the treatment of systemic diseases, these chelators need to cross the blood–brain barrier (BBB). Deferoxamine, a hexadentate ligand with high affinity for Fe(III), is capable of inhibiting amyloid formation *in vitro (* 23). However, it has difficulty in crossing the BBB when administered orally and also has side effects (24, 25). Clioquinol, a lipophilic binder, has been used successfully in phase II trials in moderate AD, but it is not iron selective and has significant side effects (26). Bearing this in mind, developing a chelator for treating neurodegenerative diseases, especially in individuals with MCI and T2DM, which is effective, easy to administer, and free of side effects, presents a considerable challenge.

Phytate (myo-inositol hexaphosphate, IP6) is a natural compound present in seeds (e.g., cereals, legumes, and nuts) as a calcium–magnesium salt (phytin) (27, 28). It possesses the ability to chelate various divalent and trivalent cations, including iron (27, 29–31) with a higher affinity than commonly used chelating agents like EDTA, deferoxamine, or deferiprone (30). This suggests that the consumption of IP6 could reduce iron bioavailability, and IP6 is able to cross de BBB (28, 32, 33). Moreover, in a study where we evaluated the relationship between dietary phytate (34), mineral status, and phytate levels in the body, it was observed that the amount of iron found in the brains of rats fed phytate diets was significantly lower than that found in rats fed non-phytate diets. Recently, an observational study has reflected that phytate intake was positively associated with cognitive function (35). Our group has shown that IP6 prevents the formation of pathological calcifications *in vivo*, such as calcium kidney stones (36, 37), sialolithiasis (38), and vascular calcification (39, 40), and protects against osteoporosis (41). In animals and cell models, phytate could also provide protection against cancer (33) and Parkinson disease (42). In a recent clinical trial with high safety levels, we have shown that daily phytate intake for 3 months is capable of reducing the levels of advanced glycation end products in T2DM patients (40). To our knowledge, no randomized clinical trial with IP6 supplementation and cognitive impairment have been carried out so far.

Our hypothesis is that phytate, due to its ability to inhibit the formation of AGEs and to chelate transition metals, will improve cognitive ability and delay the progression of mild cognitive impairment to dementia in patients with T2DM and MCI. It will also slow/prevent/mitigate the accumulation of iron in the CNS and the changes associated with neurodegeneration both in the CNS and in the retina. Likewise, changes in the biochemical markers associated with neurodegeneration are to be expected.

## 2 Objectives

### 2.1 Main aims

The main aims of this study are as follows:

1. To evaluate the effect of daily phytate intake on cognitive ability and compare it with the placebo group.
2. To determine the effect of daily phytate intake on morphological changes and iron storage associated with cognitive impairment in diabetes by magnetic resonance imaging (MRI).

### 2.2 Secondary aims

The secondary aims of this study are as follows:

1. To evaluate the effect of daily phytate intake on the evolution of retinal neurodegeneration parameters and diabetic retinopathy.
2. To determine the effect of daily phytate intake on the evolution of plasmatic biomarkers associated with cognitive decline and aging.
3. To assess the effect of daily phytate intake on body composition (using bioimpedance vector and muscle ultrasound) and grip strength (by dynamometry).

## 3 Experimental design

### 3.1 Design

The PHYND trial is a two-arm, double-blind, placebo-controlled study designed to evaluate the effect of daily phytate supplementation on the progression of mild cognitive impairment, brain iron storage, and retinal involvement in patients with type 2 diabetes. Patients diagnosed with MCI, and whose consumption of phytates is in the low/moderate range, will be recruited by the Endocrinology and Nutrition Service of the Son Llátzer University Hospital. The treatment will be diet modification: Arm 1 will take capsules of phytate dietary supplement three times a day (in the form of Lit-Control pH Balance) compared to Arm 2, which will take placebo capsules three times a day.

The present protocol was elaborated following the Standard Protocol Items: Recommendations for Intervention Trials (SPIRIT) 2013 Statement (43). Reporting of the study will follow the CONSORT statement recommendations on RCTs (44). **Table 1** summarizes all items included in the trial registry, as suggested by the World Health Organization (WHO, 2018) (45).

**Table 1:** Data collection by visits.

	Initial evaluation	Initial evaluation	Start	Continuation visit	Continuation visit	Continuation visit	Continuation visit	Last visit	Follow-up
*Weeks number*	−8	−4	0	4	14	28	42	56	65
*Informed consent*									
*Inclusion & Exclusion criteria*									
*Questions about health and disease*									
*Physical examination*									
*Blood pressure and heart rate measurement*									
*ApoE genotype*									
*LUES serology*									
*Food frequency questionnaire*									
*Medical history evaluation*									
*Grip Strength*									
*MOCA questionnaire*									
*SAGE questionnaire*									
*Magnetic Resonance Imaging*									
*Ophthalmologic evaluation*									
*Beck questionnaire*									
*Rey figure*									
*Pittsburg Sleep Quality Index*									
*2-h urinary phytate determination*									
*Body composition evaluation*									
*Lab test*									
*AD biomarkers determination (TNF-alpha, IL-1B, IL-6, NF, t-tau, p-tau 181, Ab-42, Ab-40)*									
*AGE and RAGE determination*									
*Adverse effect evaluation*									
*Pill counting*									

### 3.2 Baseline variables

Variables to be measured to evaluate inclusion criteria are the following:

1. Diagnosis of MCI: cognitive screening for MCI will be performed using the Montreal Cognitive Assessment Test (MoCA) and the Self-Administered Gerocognitive Test (SAGE). We stratified MoCA results by race/ethnicity and education level before applying a cutoff value for the MoCA score to achieve more accurate cutoffs as suggested by Milani et al. (2018). “Mild cognitive impairment” will be considered when SAGE<17 (46) and MoCA-adjusted score (47) corresponds to an MCI.
2. Neuropsychological test (NPT): in patients who score MoCA corresponds to a MCI considering race and years of education, a cognitive assessment will be carried out using the following neuropsychological tests (NPT): SAGE, digit and symbol substitution test (DSST), line test in version A and B (TMT-A and TMT-B), Rey Auditory-Verbal Learning Test (RAVLT), Rey–Osterrieth Complex Figure Copy and Recall (ROCF), Boston Naming Test (BNT), and phonetic and semantic verbal fluency will also be tested. Through these tests, we will examine the following domains: visual scanning and processing speed (DSST, TMT-A), divided attention and executive function (TMT-B and verbal fluency), learning, retention memory and verbal recall (RAVLT), constructive praxia (including motor skills), visual memory (ROCF), and language (BNT). All these tests are validated in Spanish language. NPTs will not be performed unless the diagnosis of MCI with MoCA is established.
3. Phytate dietary questionnaire: a score ≤5 will be considered low/moderate phytate consumption (48).
4. Apolipoprotein E (APOE) genotype in serum samples.

### 3.3 Participants

#### 3.3.1 Inclusion criteria

Those aged over 60 years, with a diagnosis of mild cognitive impairment, according to the MoCA and SAGE, low/moderate phytate intake, T2DM evolution of over 5 years, no history of cerebrovascular accident or neurodegenerative disease, no acute myocardial infarction or heart failure during the previous 6 months, no severe pre-proliferative retinopathy or proliferative retinopathy, and those who have signed the informed consent will be included.

#### 3.3.2 Exclusion criteria

Those with illnesses or disorders that could affect deterioration, safety or adherence, immuno-suppressants, epilepsy, neoplasia in the previous 5 years, alcohol or drug abuse, anti-Parkinsonian or anticonvulsant medication, iron metabolism pathologies such as hemochromatosis and blood disorders requiring transfusions, skin neoplasia (history of cancer during the 5 years prior to the day of screening) with the exceptions of basal cell and squamous cell carcinoma of the skin and any carcinoma *in situ*, and those who are participating in any another clinical trial will be excluded.

#### 3.3.3 *Patient withdrawal criteria*

The investigator must withdraw patients from the study when at least one of the following circumstances occurs: death, withdrawal of informed consent, and loss to follow-up. The reason and date of withdrawal from the study must be included in the Data Collection Notebook (DCN).

### 3.4 Sample calculation and randomization

There are no data on the effects of phytate supplementation on mild cognitive impairment. The sample size has been calculated based on other dietary intervention studies on MCI considering an alpha risk of 0.05 and a statistical power of 0.8 (49). Bearing in mind that we anticipate that there will be a difference of 30% in the progression of cognitive deterioration between those assigned diet 1 and those assigned diet 2, for a bilateral hypothesis test, a power of 80%, an alpha of 0.05, and a percentage of losses of 15%, we would obtain a minimum sample of 45 T2DM patients. Randomization will be 1:1 using fixed size stratified blocks. The stratification factors will be APOE genotype, age (75 years), gender, duration of diabetes (10 years), and previous cardiovascular disease (50).

## 4 Procedure

### 4.1 Description of interventions

**Figure 1:**
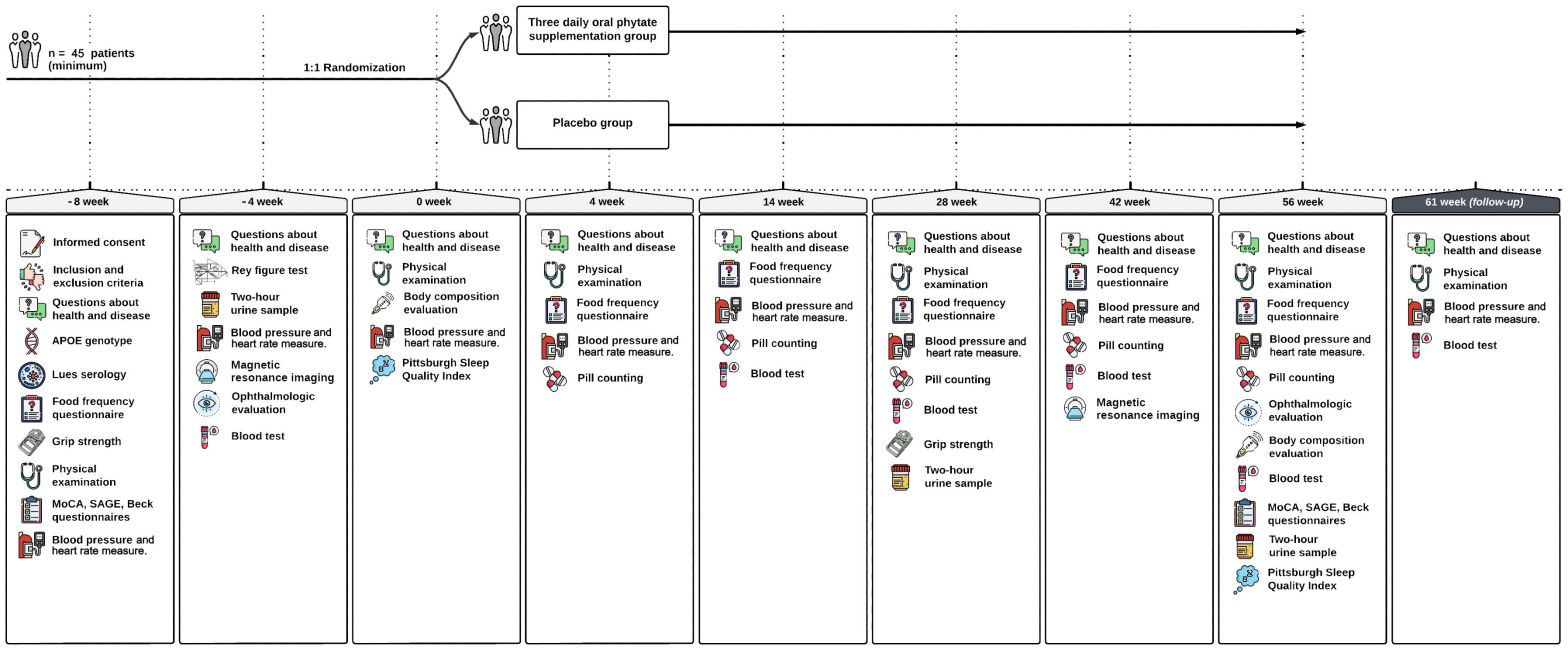
Outlines the actions to be conducted in each visit.

Patients will have nine visits between week −8 (−8) and week 56 (w56), with week 0 (w0) being the point of randomization and start of treatment. These visits will take place in the endocrinology service of the Son Llàtzer University Hospital. **Figure 1** sums up the different interventions that will take place in each visit. Furthermore, a follow-up visit will be performed at week 62 (w62).

#### 4.1.1 Visit 1 (week −8)

At the patients’ first visit (v1, w-8), inclusion criteria will be reviewed to ensure that they are eligible. To verify that the patient meets all the criteria deemed necessary to participate, the following procedures will be carried out:

- They will be informed about the different aspects of the study and given the information sheet so that they can read it carefully.
- They will be asked to read and sign the informed consent document.
- Questions about health and disease will be carried out.
- They will be asked questions about their medical history, and the researcher will check with them if they meet the requirements to participate in the project, including asking questions about their eating habits, consumption of tobacco, alcohol, and other substances.
- They will undergo screening tests for cognitive impairment and do the phytate dietary survey.
- A hand grip strength dynamometry will be performed to measure the grip strength in the dominant and contralateral hand.
- They will be asked if they are taking any other medication at the time of the project.
- The doctor will perform a complete physical examination, including taking height and weight measurements, to check their general state of health.
- Determination of APOE genotype and LUES serology will be requested.
- Their vital signs will be measured, including heart rate, blood pressure, and body temperature.
- They will be given a 2-h urine collection container to use for the next visit, and they will be scheduled for a baseline test at week −4.

#### 4.1.2 Visit 2 (week −4)

On this visit:

- Questions about health and disease will be carried out.
- They will be asked if they are taking any other medication at the time of the project.
- Their vital signs will be measured, including heart rate, blood pressure, and body temperature.
- The relevant neuropsychological tests will be completed.
- They will be scheduled for a non-contrast MRI and ophthalmological tests to take place between week −4 and week 0.

#### 4.1.3 Visit 3 (week 0)

In the case of patients who meet all the inclusion criteria and none of the exclusion criteria, the corresponding variables of the clinical history will be recorded in the DCN. On this visit:

- Questions about health and disease will be carried out.
- They will be asked if they are taking any other medication at the time of the project.
- The doctor will perform a complete physical examination, including taking height and weight measurements, to check their general state of health.
- Their vital signs will be measured, including heart rate, blood pressure, and body temperature.
- Body composition assessment will be carried out using vectorial bio-impedance analysis (BIA) and Nutritional Ultrasound to measure the diameter of the rectus femoris of the quadriceps and preperitoneal visceral adipose tissue (51).
- Non-contrast MRI and ophthalmological tests will take place between week −4 and week 0.
- Pittsburg Sleep Quality Index will be administered (52).

These patients will be randomly assigned to one of two treatment arms:

- Arm 1: 56 weeks with phytate dietary supplement
- Arm 2: 56 weeks with placebo

Diets will consist of:

*-* Treatment with phytate: patients will continue with their usual diets (low/moderate in phytate), and in addition, they will ingest three capsules a day (breakfast, lunch, and dinner) of a dietary supplement of 300 mg/sachet of phytate (in the form of phytin) over the course of 56 weeks. The doctor will give them the correct number of capsules to last until the next visit. At the next visit, a blood test will be requested (in line with usual clinical practice), and a 2-h urine sample will be collected again to determine the urinary phytate concentration at weeks 0, 28, and 56.
*-* Placebo treatment: patients will continue with their usual diets (low/moderate in phytate) and, in addition, will ingest three placebo capsules a day (breakfast, lunch, and dinner) over the course of 56 weeks. The doctor will give them the correct number of capsules to last until the next visit. At the next visit, a blood test will be requested (in line with usual clinical practice), and a 2-h urine will be collected again to determine the urinary phytate concentration at weeks 0, 28, and 56.

#### 4.1.4 Visits 4 (week 4), 5 (week 14), 6 (week 28), and 7 (week 42)

On these visits:

- Questions about health and disease will be carried out.
- They will be asked if they are taking any other medication at the time of the project.
- Safety will be reviewed. Any of the following outcomes will be recorded as serious adverse events: death, hospitalization of more than 24 h, persistent disability, and any other results that endanger the life of the subject. Episodes of clinically significant iron deficiency or anemia and episodes of hypoglycemia <54 mg/dl and level III hypoglycemia (that is, requiring the assistance of a third party) will be collected with particular interest.
- The doctor will perform a complete physical examination, including taking height and weight measurements, to check their general state of health at weeks 0 and 28.
- Their vital signs will be measured, including heart rate, blood pressure, and body temperature.
- At week 28, a urine sample will be collected.
- At weeks 14, 28, and 42, blood tests will be performed.
- Patients will be given a dietary survey to assess their adherence to the low phytate diet.
- Remaining supplement capsules will be collected and counted, and the correct number of capsules will be given to last until the next visit.
- Patients will be given a 2-h urine collection container that must be brought to their next visit at week 28.
- The final visit will be scheduled, where a blood test will be performed in line with usual clinical practice.

#### 4.1.5 Visit 8 (week 56)

At this end-of-treatment visit:

- Safety will be reviewed, as above.
- Neuropsychological and cognitive deterioration tests will be completed again.
- Urine samples will be collected, and a blood test will be performed.
- A dietary survey to assess patient adherence to the low phytate diet will be completed.
- Any surplus supplement capsules will be collected, counted, and accounted for.
- The doctor will perform a complete physical examination, including taking height and weight measurements, to check the patient’s general state of health.
- Vital signs will be measured, including heart rate, blood pressure, and body temperature.
- A non-contrast MRI and ophthalmological tests will be performed this week.
- Body composition assessment will be carried out using vectorial BIA and Nutritional Ultrasound to measure the diameter of the rectus femoris of the quadriceps and preperitoneal visceral adipose tissue (51).
- Pittsburg Sleep Quality Index will be administered.

#### 4.1.6 Visit 9 (week 62)

In the follow-up visit:

- Questions about health and disease will be carried out.
- Their vital signs will be measured, including heart rate, blood pressure, and body temperature.
- Complete physical examination will be performed.
- A routine blood test will be carried out.

### 4.2 Manufacture of phytate and placebo

The phytate supplement and the placebo were purchased from the company Devicare S.L. The phytate will be administered in the same pharmaceutical form as the Lit-Control^®^ pH Balance dietary supplement with national code (CN) 183677.9. The placebo treatment will be manufactured at ELABORADOS DIETÉTICOS S.AU., as a sub-contracted manufacturing plant with Good Manufacturing Practice certification.

## 5 Results

### 5.1 The main variables (according to the main objectives) will be the following:

1. Progression of cognitive impairment as a dichotomous variable defined as a score decrease >1 SD in MoCA or in any of the cognitive domains studied [visual scanning and processing speed (DSST, TMT-A), divided attention and executive function (TMT-B and verbal fluency), learning, retention memory and verbal recall (RAVLT), praxia, visual memory (ROCF) and language (BNT)] at week 0 and week 56.
2. Differences in the quantification of the iron deposit assessed by nuclear magnetic resonance (NMR) (T2W (R2* and R’2; QSM) (General Electrics, Signa Explorer 1.5T) at week 0 and week 56. We quantify iron content in specific brain areas using magnetic resonance imaging (MRI) techniques such as quantitative susceptibility mapping (QSM) or R2* relaxometry (53). QSM describes the magnetizability of a material to an applied magnetic field and is a substance-specific value (54). Iron causes MRI images to darken at a rate proportional to the iron load, with the half-life of this darkening defined as T2* (55). The rate of darkening, designated as R2*, is the reciprocal of T2* and is proportional to the iron content of the tissues (55). MRI scanning estimates tissue iron concentration both by gradient echo imaging, which provides T2*, and spin echo imaging, which provides T2, the reciprocal of R2 (55). Novel NMR techniques allowed an improvement of tissue metal mapping methods and also the understanding of the ferroptosis mechanisms (53, 55).

### 5.2 The secondary variables (depending on the secondary objectives) will be the following:

1. Quantitative differences detected by the different cognitive tests (W0 and W56).
2. Morphological differences in the MRI without contrast (W0 and W56): degree of atrophy of the medial temporal lobe (Scheltens score) (56), periventricular intensity and hyperintense lesions in deep white matter (Fazekas scale) (57), and number of lacunar infarcts detected.
3. Differences in retinal neurodegeneration parameters and evolution of diabetic retinopathy (W0 and W56).
4. Retinal function: best-corrected visual acuity (BCVA) and macular perimetry.
5. Structural evaluation of the retina:
  - Seven-field retinography and staging of diabetic retinopathy according to the Early Treatment Diabetic Retinopathy Study (58). Image of the disk-centered fundus, used to study the caliber and morphology of the vessels.
  - Wide-field images with and without fluorescein to examine the peripheral retina and the degree of hypoperfusion (59) ^(^ 60 ^),^.
  - Optical coherence tomography (OCT-SD) to assess neurodegeneration and macular edema parameters such as retinal nerve fiber layer (RNFL) thickness, ganglion cell layer (GCL) thickness, and choroidal thickness will be collected (61).
  - OCT-angiography: to evaluate in detail the capillary plexuses of the macula, both the vessel density (VD), perfusion density (PD) in both superficial capillary plexus, and the foveal avascular zone (FAZ) will be collected (60).
6. Effect on AD biomarkers (W0 and W56):
  - Inflammation mediators: TNF-a, IL-1b, and IL-6 (Multiplex)
  - Neuronal injury markers: neurofilament (NF) (SimoaTM)
  - AD biomarkers: t-tau, p-tau181, Ab42 and Ab40 (ELISA)
7. AGEs and RAGEs (ELISA)
8. Differences in sleep quality reported by Pittsburg Sleep Quality Index.
9. Differences body composition assessed with vectorial bio-impedance analysis and Nutritional Ultrasound to measure the diameter of the rectus femoris of the quadriceps and preperitoneal visceral adipose tissue.
10. Effect on hand grip strength dynamometry in the dominant and contralateral hand.

### 5.3 Other variables

Other variables include the following:

1. As the cognitive status of participants can be influenced by depression and educational level, this information will be collected, and a Beck Depression Inventory test will be performed to assess depressive symptoms (W0 and W56).
2. Laboratory parameters (W0, W14, W28, W42, W56, and W62). Glucose, HbA1c, liver profile, iron, ferritin, transferrin, transferrin saturation, calcium, phosphate, 25-(OH)-vitamin D (W0, W28, W56, and W62), PTH (W0, W28, and W56), creatinine, profile lipid, complete blood count, microalbuminuria/creatinine (W0, W28, and W56), and cystatin C, insulin, and hsPCR (W0 and W56). FIB-4 will be calculated.
3. Urinary phytate in 2 h urine (W-4, W28, and W56) (62).
4. Clinical history and results of physical examination: age, sex, race, educational level, date of diagnosis of T2DM, height (W-8, W0, and W56), weight, BMI, blood pressure, heart rate, waist circumference (W0, W14, W28, W42, W56, and W62), hypertension, cardiovascular disease, diabetic retinopathy, alcohol and tobacco consumption, history of polyneuropathy (W-8, W0, and W56), treatment of T2DM, and intake of other medications (W0, W14, W28, W42, W56, and W62).

### 5.4 Statistical analysis

For the analysis of primary and secondary objectives, differences within groups will be assessed using ANOVA for repeated measures, and difference between groups will be assessed using ANCOVA for repeated measures. Qualitative variables will be analyzed using the chi-square test or the McNemar’s test. The analysis will be adjusted for confounding variables. With regard gender dimension, women have a higher risk of dementia; therefore, an additional analysis will be carried out, stratifying results by sex. A value of p<0.05 will be considered an indicator of a significant difference. The analysis will be performed using SPSS software (IBM Corp., version 24.0).

### 5.5 Ethical considerations

The clinical researchers involved in the trial have a valid certificate of Good Clinical Practice. The study will adhere to law 14/2007 on biomedical research, the principles of the Declaration of Helsinki, and the Council of Europe Convention on Human rights and Biomedicine. Our protocol has been approved by the Ethics and Clinical Research Committee of the Balearic Islands (CEI-IB) with number “IB4719/21”. The study will be carried out according to the standards of Good Clinical Practice and Guidelines of the International Conference on Harmonization (ICH).

## 6 Safety

The treatment that is used, Lit-Control^®^ pH Balance, has been developed with ingredients that have been internationally recognized as safe. No incidents have been reported by its consumers since the beginning of its commercialization on said date. However, in case that a possible serious adverse event was to be detected and recorded, the treatment would be suspended and the investigator would proceed as follows:

- The sponsor would be immediately informed of all adverse events. Immediate notification must be followed by timely detailed written reports. The immediate and follow-up report must identify subjects by a unique code assigned to the trial subject and not by the subject’s name, personal identification numbers, or subject’s address. The investigator must also comply with the legal regulations regarding the notification of serious and unexpected adverse events to the relevant authorities and to the CEI-IB.
- When the researcher notifies a death, he must provide the promoter and the CEI-IB with all the complementary information that they request (e.g., the autopsy report and the most recent medical reports). An adverse event (AE) will be considered any adverse health incident that occurs in a patient who has received a treatment, even where there is not necessarily a causal relationship with said treatment. An adverse event (AE) can therefore be any unfavorable and unintended sign (including an abnormal laboratory finding), symptom, or disease temporarily associated with the use of treatment, whether or not related to it.

According to current regulations, “serious adverse events” will be considered as “any adverse reaction that causes death, may be life-threatening, requires hospitalization of the patient or prolongation of existing hospitalization, causes significant or persistent disability or invalidity, or constitutes a congenital anomaly or birth defect. Those suspected adverse reactions that are considered important from a medical point of view, even if they do not meet the above criteria, such as those that put the patient at risk, or require intervention to prevent any of the above outcomes, will also be treated as serious, as will all suspicions of transmission of an infectious agent through a medication (RD 1090/2015, art. 22.1.d “Serious adverse reaction”)”.

## 7 Validity and reliability

The current study will provide important information on the effect of oral phytate supplementation on cognitive impairment progression, cerebral iron deposition, and diabetic retinopathy on patients living with T2DM. The PHYND study will include a control sample of participants that will receive placebo. The free-living conditions and the study design will allow us to obtain robust results. Internal validity will be warranted by a randomization process based on allocation sequence generation, blinded to the PI and staff involved in the intervention. Moreover, the data analyst and the PI will be blinded to patient allocation to reduce biases in the evaluation of the intervention. Lastly, the interventions are based on the latest scientific evidence.

## 8 Discussion

The present study will provide information on the efficacy of an oral phytate supplementation intervention (≈1g/day) to reduce cognitive impairment progression, cerebral iron deposition, and diabetic retinopathy on patients living with T2DM. There is no specific treatment available to reduce cognitive impairment progression on patients with T2DM. Diets known to be high in phytate are associated with lower cognitive decline (33). So far, there is no randomized clinical trial assessing the effect of phytate intake in cognitive decline and neurodegenerative disease. Our work will be the first one in assessing the effects of oral phytate supplementation in amounts corresponding to a healthy and balanced diet rich in nuts and legumes (≈1g/day) on cognitive impairment progression in outpatients conditions.

The PHYND study presents some limitations. First, it focuses on patients with T2DM and MCI, potentially restricting the generalizability of results to broader populations. Second, ensuring participant compliance with the daily phytate supplementation regimen is crucial, yet despite monitoring mechanisms like pill counts and urinary phytate level assessments, inconsistent adherence may impact result interpretation. Third, despite rigorous blinding procedures and the use of validated outcome measures to minimize placebo effects, improvements related to the placebo effect within the placebo group are still possible, which could influence the interpretation of study outcomes. The most important limitation will be patient adherence to the treatment and the study, given its length, i.e., 56 weeks of intervention. However, this issue will be mitigated by frequent follow-up, frequently telephonic reminders of the appointments, and motivational interview. Another issue is the small sample size. We anticipate a 15% drop off based on other dietary intervention studies on MCI considering an alpha risk of 0.05 and a statistical power of 0.8(49); a minimum sample of 45 T2DM patients is required. Keeping this in mind, we will recruit 60 patients per arm. Thus, reducing the probability of small sample is a limitation of our work. In addition, as the study is single center (the sample will be taken at the Endocrinology and Nutrition service of the Son Llàtzer Hospital), we are aware that it could not be possible to generalize the results obtained.

However the strengths of our work are a multidisciplinary approach, where the effect of phytate supplementation on the brain and the retina will be objectively examined using validated ophthalmic tests, magnetic resonance imaging iron measurement, evaluation of cognitive impairment through validated psychological tests, extensive laboratory tests (using the accepted laboratory parameters, inflammatory mediators, markers of neuronal injury, AD biomarkers, and advanced glycation products), and measurement of body composition and strength using nutritional ultrasound and bio-impedance vector analysis and dynamometry. In addition, as the cognitive status of participants can be influenced by depression, educational level and quality, and quantity of sleep, this information will be collected with validated tests. Phytate levels will be monitored by measuring adherence to diet supplementation and serial measurement of urinary phytate levels.

Finally, the results of our study could contribute to better nutritional strategies for the management of cognitive impairment and diabetic-related complications. Given the increasing prevalence of T2DM and cognitive impairment, determining interventions that could prevent or reduce diabetes-related complications and reduce the progression of cognitive impairment could have a great impact on the lives of our population.

## Ethics statement

The studies involving humans were approved by Ethics and Clinical Research Committee of the Balearic Islands (CEI-IB) with number “IB4719/21”. The studies were conducted in accordance with the local legislation and institutional requirements. Written informed consent for participation was not required from the participants or the participants’ legal guardians/next of kin in accordance with the national legislation and institutional requirements.

## Author contributions

AP: Conceptualization, Data curation, Investigation, Validation, Writing – original draft, Writing – review & editing. PS: Conceptualization, Data curation, Funding acquisition, Investigation, Methodology, Project administration, Resources, Visualization, Writing – original draft, Writing – review & editing. MT: Conceptualization, Investigation, Methodology, Project administration, Validation, Writing – review & editing. JN: Conceptualization, Investigation, Methodology, Writing – review & editing. FG: Funding acquisition, Investigation, Methodology, Resources, Validation, Visualization, Writing – review & editing. AEsp: Investigation, Methodology, Validation, Visualization, Writing – review & editing. AEst: Conceptualization, Methodology, Validation, Writing – review & editing. ER: Investigation, Methodology, Writing – review & editing. GA: Investigation, Methodology, Writing – review & editing. MR: Investigation, Methodology, Writing – review & editing. JR: Investigation, Methodology, Writing – review & editing. IG: Investigation, Methodology, Writing – review & editing. OS-S: Investigation, Methodology, Writing – review & editing. LM: Resources, Supervision, Validation, Visualization, Writing – original draft, Writing – review & editing, Conceptualization, Funding acquisition, Investigation, Methodology, Project administration.
